# Elective peripartum hysterectomy for placenta accreta: is it always a severe maternal morbidity?

**DOI:** 10.4274/jtgga.galenos.2020.2020.0102

**Published:** 2021-02-24

**Authors:** Pradip Kumar Saha, Aashima Arora

**Affiliations:** 1 Department of Obstetrics and Gynecology, Post Graduate Institute of Medical Education and Research, Chandigarh, India

To the Editor,

The American College of Obstetricians and Gynaecologists (ACOG) and the Society of Fetal Medicine (SFM) have published a document saying that records of all women needing more than 4 units of blood transfusion or intensive care unit (ICU) admission must be reviewed to identify women with severe maternal morbidity (1).

To date, there is no clear consensus as to what conditions should be termed severe maternal morbidity. ACOG and SFM have given an example list of diagnoses and complications constituting severe maternal morbidity, wherein all women undergoing emergency or unplanned peripartum hysterectomy are assumed to have severe morbidity, while planned peripartum hysterectomies for cancers are not considered to cause severe morbidity. All cases of hysterectomy for placenta accreta have been classified as severe morbidity irrespective of the number of blood transfusions or ICU admission (1).

The government of India’s operational guidelines for the “maternal near-miss” state that any woman who requires emergency hysterectomy for controlling blood loss is to be labeled as a near-miss (2). World Health Organization classifies hysterectomy due to uterine infection or hemorrhage as a critical intervention, labeling the woman as a near-miss (3). However, in all these guidelines, a planned peripartum hysterectomy done for a pre-operatively diagnosed placenta accreta where the woman remains hemodynamically stable does not find an appropriate mention.

Placenta accreta spectrum (PAS), formerly known as morbidly adherent placenta, refers to the range of pathologic adherence of the placenta, including placenta increta, placenta percreta, and placenta accreta. This is definitely a potentially life-threatening condition with an increasing incidence all over the world, secondary to the rising caesarean section rate. The recommended management is a planned caesarean hysterectomy before term, where no attempt must be made to separate the placenta from the uterus. Though the exact timing of scheduled delivery remains controversial, with ACOG recommending planned surgery at 34 to 36 weeks, Royal College of Obstetrics and Gynecology at 35 to 36+6 weeks and SFM at 34 to 37 weeks, the basic principle is to balance the benefit of fetal lung maturity with a risk of excessive haemorrhage when the surgery is performed in an emergency setting (4,5,6).

Optimal pre-operative preparation, adoption of a multidisciplinary approach and minimizing blood loss intraoperatively are the most critical steps in the management of PAS. Many surgical approaches have been suggested over time with variable benefits. One such approach is separating the bladder up to the cervico-vaginal junction prior to uterine incision so that the aberrant blood vessels traversing between uterus and bladder are ligated and divided. We have reported significantly reduced blood loss with this technique, minimizing the need for massive blood transfusion. None of the women out of 12 cases performed over 17 months required ICU stay or had bladder or ureteric injury (7).

When performed in tertiary centers with all necessary preparation by a multidisciplinary expert team, this surgery may have significantly reduced morbidity. Thus the present recommendation of considering all peripartum hysterectomy performed for placenta accreta as a near miss may not always be appropriate. In our opinion peripartum hysterectomy performed for placenta accreta should be further classified in order to properly categorize the cases which actually are near misses.
